# Yoga as a Therapeutic Intervention

**DOI:** 10.1155/2012/174291

**Published:** 2012-11-11

**Authors:** Arndt Büssing, Sat Bir S. Khalsa, Andreas Michalsen, Karen J. Sherman, Shirley Telles

**Affiliations:** ^1^Department of Quality of Life, Spirituality, and Coping, Center of Integrative Medicine, Faculty of Healthy, Witten/Herdecke University, 58313 Witten, Germany; ^2^Division of Sleep Medicine, Brigham and Women's Hospital, Harvard Medical School, Boston, MA 02215, USA; ^3^Department of Internal and Complementary Medicine, Immanuel Hospital Berlin and Institute of Social Medicine, Epidemiology & Health Economics, Charite-University Medical Center, 14109 Berlin, Germany; ^4^Group Health Research Institute, Group Health Cooperative, Seattle, WA 98101, USA; ^5^Patanjali Research Foundation, Uttarakhand, Haridwar 249405, India

Yoga is an ancient mind/body practice, which originated in the Indian subcontinent, that promotes overall health and well-being. Yoga's ultimate goal is the achievement of a state of unified consciousness. Historically, the practice of yoga included eight steps towards spiritual emancipation and included practices involving physical postures, breathing techniques, and meditation. Today, yoga is very popular in the West amongst the general public. Numerous modern schools or styles of yoga exist (i.e., Iyengar, Ashtanga, Viniyoga, Bikram, Sivananda, Kripalu, Kundalini, Integral, among others), most of which are forms of traditional Hatha yoga, each with their own distinct priorities in terms of the balance of inclusion of spiritual and physical practices, with some styles focusing more exclusively on physical postures despite the historical focus of yoga on inner development. Research on the psychophysiological benefits of yoga and meditation practice has revealed benefits in physical, mental, and emotional self-regulation. Demonstrated improvements in stress, anxiety, mood, and physical health and well-being have proven useful for therapeutic purposes, and this has led to the popular implementation of yoga as a primary or adjunctive therapy.

Despite several clinical studies and systematic reviews on the effects of the different yoga styles, further research is required to clarify yoga's value as an intervention. With respect to yoga's value for specific indications and disorders, most studies report positive effects in favor of yoga interventions, although there are a variety of effect sizes observed. The degree of clinical benefit of yoga in any specific research trial may depend on a variety of factors including participant characteristics (age, gender, health status, etc.), diagnoses and study entry criteria, yoga intervention characteristics (e.g., yoga styles, intensities, and frequencies and durations of practice), compliance and attrition effects, and so forth. Moreover, as a relatively new field of research, most of the research trials are pilot studies, with typically small samples sizes, moderate methodological quality, and often inadequate control groups rendering the general findings tentative and in need of further research validation. 

For this special issue, we have invited investigators to submit original research articles and systematic reviews/meta-analyses on the clinical effects of yoga intervention programs. The special issue starts with a summarizing overview of published literature reviews on the clinical effects of yoga interventions on physical and mental health, and it continues with a wide range of scientific contributions, addressing specific aspects of a colorful field of research. Of course we are aware of the many unanswered questions and the methodological flaws and limitations of several of these studies, yet we believe that the papers of this special issue will shed light on a fascinating and expanding field within mind-body medicine that is rich with promising findings.

*Arndt Büssing* *Arndt Büssing* *Sat Bir S. Khalsa* *Sat Bir S. Khalsa* *Andreas Michalsen* *Andreas Michalsen* *Karen J. Sherman* *Karen J. Sherman* *Shirley Telles* *Shirley Telles*

